# An Unusual Case of Blastomycosis and Severe Lung Necrosis in a Hmong Woman With Preexisting Liver Cirrhosis

**DOI:** 10.7759/cureus.17406

**Published:** 2021-08-24

**Authors:** Leilani Hernandez, Pinky Jha, Peter Sohnle

**Affiliations:** 1 Internal Medicine, Medical College of Wisconsin, Wauwatosa, USA; 2 Division of Infectious Diseases, Department of Medicine, Medical College of Wisconsin, Wauwatosa, USA

**Keywords:** hmong, blastomycosis, liver disease, splenic artery embolization, liver cirrhosis

## Abstract

Predisposing factors that lead to blastomycosis remain unknown, although like other fungal infections, blastomycosis is an opportunistic infection. Here, we report on an unusual presentation in a Hmong woman with preexisting liver disease. This case highlights genetic and medical factors that may increase susceptibility to blastomycosis.

## Introduction

Blastomycosis is known as the “great pretender” due to its wide range of clinical manifestations [1]. It occurs after inhalation of *Blastomyces dermatitidis* spores. Prior outbreaks in Wisconsin have shown an association with outdoor activities near rivers and ponds, construction, or digging up soil; however, there may also be sporadic infections not clearly associated with outdoor activities [1-3]. In endemic regions, blastomycosis may be more common and/or severe in patients with AIDS, solid organ transplants, or those receiving tumor necrosis factor (TNF) inhibitor therapy [1]. However, much remains unknown about pertinent medical risk factors that influence disease severity of these infections. Here, we report on an unusual presentation of blastomycosis in a Hmong woman with preexisting hepatic cirrhosis whose infection resulted in severe lung necrosis and cavitation.

## Case presentation

A 45-year-old Hmong woman presented to the Emergency Department with a three-week history of productive cough, progressive shortness of breath, and ascites. Medical history was significant for liver disease related to recurrent pyogenic cholangitis, a condition that is common in Southeast Asia where this patient spent her childhood before emigrating to the United States [4]. Her liver disease was categorized as Child-Pugh class B cirrhosis, with recurrent ascites, spontaneous bacterial peritonitis, esophageal varices, chronic portal vein thrombosis, portal hypertension, and splenomegaly. For the latter, she had had splenic artery embolization two years before the present episode of blastomycosis; this procedure had resulted in a chronic left pleural effusion. 

On admission, she endorsed fevers and chest pain but denied sore throat, congestion, hemoptysis, headaches, rash, sick contacts, and recent travel. Her temperature was 99.9 degrees Fahrenheit, pulse was 120 beats per minute, respiratory rate was 19 breaths per minute, and oxygen saturation was 95% on room air. Lung examination revealed rhonchi throughout all lung fields. Chest x-ray showed bilateral perihilar and basilar opacities greater on the left side. Paracentesis was done and was negative for infection. Respiratory therapy and antibiotics were initiated for treatment of rhonchi. She was started on ceftriaxone and azithromycin on admission, with vancomycin added the next day. Despite antibiotics, she continued to have fevers, cough, and shortness of breath. Per Infectious Disease consultation, a CT scan and cultures were obtained. Other infectious workups including QuantiFERON-TB and COVID-19 testing were negative. Chest CT revealed complete consolidation of the left lower lobe and early central cavitary changes. Itraconazole was initiated three days after admission when sputum samples revealed broad-based budding yeast. Testing of urine samples revealed levels of Blastomyces antigens above the upper limit of detection. Amphotericin B was added one day later for worsening respiratory status and persistent tachycardia. She was intubated in the ICU six days after admission for impending respiratory failure.

**Figure 1:**
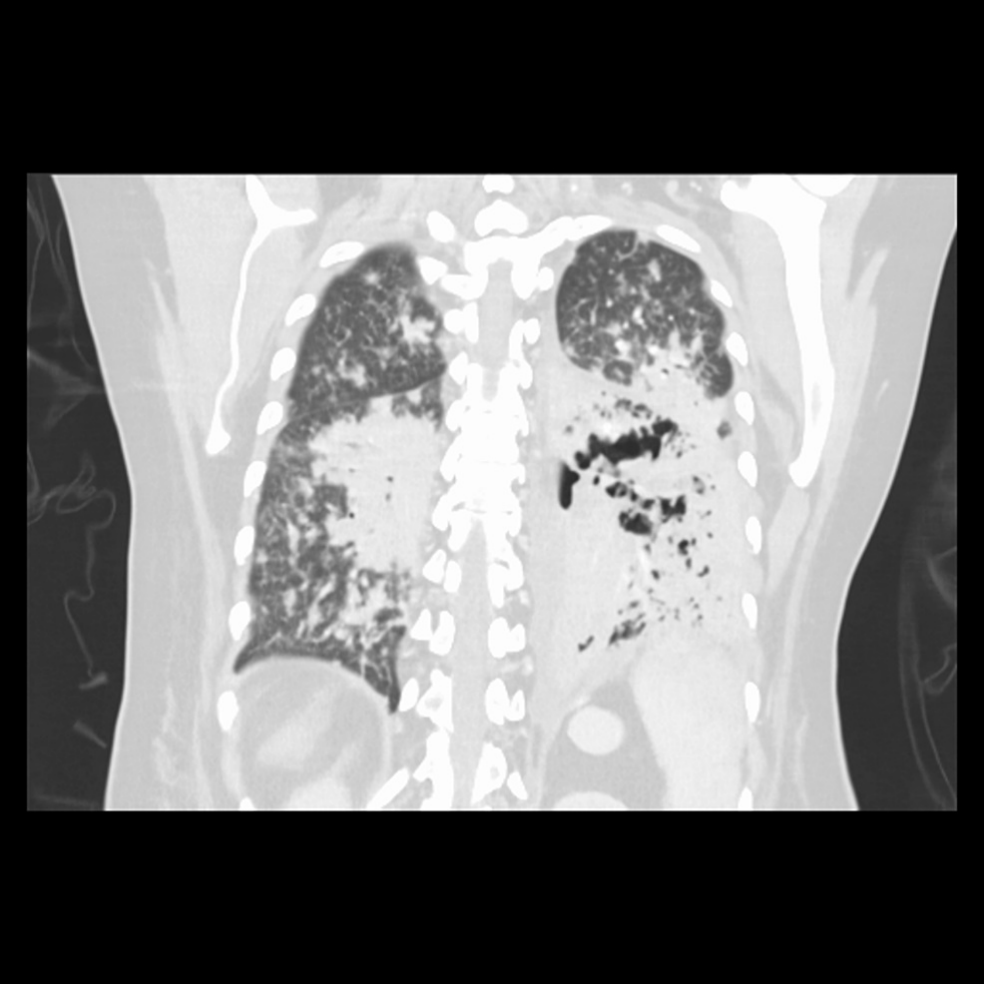
Coronal Maximum Intensity Projection CT Chest Diffuse scattered consolidative and nodular densities primarily with near complete consolidation of the left lower lobe. Imaging findings are highly suggestive of multifocal pneumonia. Fungal infection is within the differential given the appearance.

In the ICU, she often needed suctioning five to six times per hour to prevent desaturation. Bronchoscopy demonstrated a great deal of necrotic debris in her airways. Bronchoalveolar (BAL) fluid obtained during bronchoscopy revealed very high fungal burden. A second CT revealed a necrotic left lower lobe and new cavitation in the right middle lobe and left upper lobe (Figure 1).

She was continued on amphotericin B, and it was found that her respiratory status improved after therapeutic bronchoscopies to remove debris; one of these included endobronchial delivery of amphotericin B and urinary trypsin inhibitor to the left lower lobe. She eventually improved, was extubated, and was transferred back to the hospital floor in stable condition (temperature was 97.5 degrees Fahrenheit, respiratory rate was 16 breaths per minute, oxygen saturation was 92% on room air). Intravenous amphotericin B was discontinued on the floor, and final recommendations from Infectious Disease were to continue itraconazole 300 twice daily for six months. After a month-long hospital stay, she was discharged on oral itraconazole to be followed up by Infectious Disease as an outpatient.

## Discussion

Her presentation was notable for very high organism burden as evidenced from the BAL fluid, extensive lung necrosis, and excessive amounts of debris in the airways. It has already been established that Hmong are at an increased risk for blastomycosis; an investigation of a large outbreak in 2009 to 2010 in Marathon County, Wisconsin, demonstrated a clear link to Hmong ethnicity [5]. Of 55 blastomycosis cases in that series, the incidence rate among Asians was 12 times that of non-Asians, with a high proportion of Asian case-patients being Hmong [5]. Wisconsin is also an area known to be endemic for blastomycosis and has a large population of Hmong residents [6].

As shown by the studies of Merkhofer et al [7], Hmong appear to be more susceptible to blastomycosis due to genetic differences in cytokine production related to the interleukin (IL)-6/IL-17 pathway. In this work, whole genome sequencing of Hmong volunteers with confirmed blastomycosis identified 25 polymorphisms near the IL-6 locus. In addition, immortalized B lymphoblastoid cell lines from Hmong donors were found to produce significantly less IL-6 compared to cells from patients of European ancestry. A murine model using IL6^−/−^ mice demonstrated that these animals produced significantly fewer T helper 17 (Th17) cells in response to *B. dermatitidis* antigens and also had a significantly higher blastomycosis loads when challenged by experimental blastomycosis infection. The results of this model support that Hmong, who produce less IL-17 and thus less IL-17, are more susceptible to blastomycosis.

She also had a chronic cough that started shortly after splenic artery embolization two years beforehand. This procedure was used to treat focal left upper quadrant pain and decreased oral intake caused by marked splenomegaly related to the hepatic cirrhosis. Splenectomy was not an option due to her liver disease. After this procedure was performed, she was hospitalized for shortness of breath due to left pleural effusion. After discharge, records from follow-up visits noted a chronic cough that was attributed to the left pleural effusion. Her history of splenomegaly, splenic artery embolization, and chronic left pleural effusion could explain why her infection was most severe in the left lower lobe. The necrotic left lower lobe was an atypical finding in that blastomycosis has a tendency to affect the upper lobes, and cavitary lung disease is less common in blastomycosis than in histoplasmosis and tuberculosis [8]. Complications of splenic artery embolization are more common in pretransplant patients [9], and these complications do include pneumonia. It should be noted that she was offered a liver transplant but declined.

This patient’s hepatic cirrhosis secondary to recurrent pyogenic cholangiopathy may have predisposed her to a more severe presentation of blastomycosis. Her history of hepatic cirrhosis could relate to the cavitary lesions identified on imaging. Whereas there is not a clear relationship between liver disease and severe blastomycosis, a similar case has been described in which autopsy revealed dense lung consolidation, necrosis of the upper lobes, and hepatic cirrhosis [10]. A link between blastomycosis and cirrhosis might also increase vulnerability in Hmong to this type of infection. Hmong already have a predisposition to liver disease from nonalcoholic steatohepatitis, hepatitis B, and hepatocellular carcinoma [11-13].

## Conclusions

In summary, we report a case of blastomycosis with atypical findings in a Hmong woman with a medical history of hepatic cirrhosis and splenomegaly treated with splenic artery embolization. This case highlights a number of reasons why Hmong may be more susceptible to blastomycosis. 
